# Late-onset neonatal infections and bacterial multidrug resistance

**DOI:** 10.1590/1984-0462/2023/41/2022068

**Published:** 2023-05-29

**Authors:** Carmen Sulinete Suliano da Costa Lima, Hermano Alexandre Rocha Lima, Cláudia Sofia de Assunção Gonçalves e Silva

**Affiliations:** a Universidade Fernando Pessoa, Porto, Portugal.; b Harvard T. H. Chan School of Public Health, Boston, MA, USA.; c Department of Community Health, Federal University of Ceará, Fortaleza, CE, Brazil.

**Keywords:** Cross infection, Newborn, Bloodstream infections, Antibacterial drug resistance, Infecção hospitalar, Recém-nascido, Infecção da corrente sanguínea, Resistência bacteriana a antibióticos

## Abstract

**Objective:**

This study aims to describe bacterial and antimicrobial sensibilities in late-onset healthcare-associated infections (HAIs) with laboratory confirmation in a Neonatal Intensive Care Unit (NICU) of a public hospital in Ceará.

**Methods:**

This was a cross-sectional study conducted from January 2013 to December 2017. The bacterial types involved in late-onset HAIs, their sensitivity to antimicrobials, and their multidrug resistance were evaluated. The latter was classified according to the criteria revised by the Pan-American Health Organization as multidrug resistance (MDR), extended drug resistance (XDR), or pandrug resistance (PDR). The description of the variables was performed through proportions and frequency distribution depicted in tables.

**Results:**

Of the 427 patients with late-onset HAIs, 47 (11.0%) had bacterial infections confirmed by blood cultures, and 7 (14.9%) had infections caused by MDR bacteria. Among the types of bacteria, 26 (55.3%) were Gram-negative bacteria, and 21 (44.7%) were Gram-positive bacteria. Among the Gram-negative bacteria, 92.3% (n=24) showed resistance to more than one antimicrobial, especially to ampicillin (81.2%), cefepime (33.1%), gentamicin (19.4%), and piperacillin/tazobactam (17.2%). Among the MDR ones, three cases had *Klebsiella pneumoniae*, and three had *Pseudomonas aeruginosa*, classified as two MDR and one XDR, and three XDR, respectively. Gram-positive resistance to penicillin was the most common one (80.0%), and approximately half of the strains being resistant to oxacillin. Susceptibility was high to vancomycin (97.5%), but one microorganism was resistant to oxacillin and vancomycin.

**Conclusions:**

The emergence of MDR strains is a reality in NICUs, carrying the risk of therapeutic failure and requiring continuous prevention protocols aimed at minimizing the risks of contamination by bacteria with high morbidity and mortality.

## RESUMO

**Objetivo:**

Descrever as bactérias e sensibilidades aos antimicrobianos nas infecções relacionadas à assistência à saúde (IRAS) tardias com confirmação laboratorial em Unidade de Terapia Intensiva Neonatal (UTIN) de um hospital público do Ceará.

**Métodos:**

Estudo transversal, de janeiro de 2013 a dezembro de 2017. Foram avaliados os tipos de bactérias das IRAS tardias, a sensibilidade aos antimicrobianos e a multirresistência. Esta foi classificada segundo os critérios revisados pela Organização Pan-Americana da Saúde como MDR, ou multirresistência (*multidrug resistance*); XDR, ou resistência estendida (*extensively drug-resistance*); ou PDR, panresistência (*pandrug-resistance*). A descrição das variáveis foi realizada por meio de proporções e distribuição das frequências na forma de tabelas.

**Resultados:**

Dos 427 pacientes com IRAS tardias, 47 (11,0%) apresentaram infecções bacterianas confirmadas por hemoculturas, sete (14,9%) das quais foram causadas por bactérias multirresistentes. Entre os tipos de bactérias, 26 (55,3%) foram Gram-negativas e 21 (44,7%) Gram-positivas. Entre as primeiras, 92,3% (n=24) apresentaram resistências a mais de um antimicrobiano, destacando-se ampicilina (81,2%), cefepima (33,1%), gentamicina (19,4%) e piperacilina/tazobactam (17,2%). Entre as multirresistentes, três foram *Klebsiella pneumoniae* e três *Pseudomonas aeruginosa*, classificadas como duas MDR e uma XDR, e três XDR, respectivamente. A resistência das Gram-positivas à penicilina foi a mais comum (80,0%). A susceptibilidade foi alta à vancomicina (97,5%), porém uma bactéria foi resistente à oxacilina e à vancomicina.

**Conclusões:**

O aparecimento de cepas multirresistentes é uma realidade em UTIN com risco de falha terapêutica, sendo necessários protocolos contínuos de prevenção a fim de minimizar os riscos de contaminação interpessoal e ambiental por bactérias de alta morbimortalidade.

## INTRODUCTION

Late-onset healthcare-associated infection (HAI) of hospital origin in neonatology is defined as an infection acquired in healthcare services, whose diagnostic evidence occurs after the first 48 h of life in the hospitalized newborn (NB), who may or may not have laboratory confirmation by blood culture.^1,2^ NBs admitted to the Neonatal Intensive Care Unit (NICU), mainly when preterm and with very low birth weight, are exposed to many high-risk therapeutic interventions, such as the use of invasive devices and broad-spectrum antibiotics, which influence the colonization of the NB and increase the risk of infection.^3,4,5,6,7,8,9,10,11,12^ Increased survival of high-risk NBs comes with complications and, among them, late-onset HAIs stand out, often leading to death in this period.^2,13,14,15^ In NICUs, inappropriate and empirical use of several antimicrobials are the main causes of bacterial resistance, aggravated by prolonged hospitalization, the use of invasive devices, and non-adherence to isolation and safety standards, which in turn increases the risk of the emergence of infections caused by multidrug-resistant (MDR) bacteria, which can become endemic.^16–18^

The most often reported MDR bacteria in the literature are *Klebsiella pneumoniae* and *Escherichia coli*,^17^ in addition to methicillin-resistant *Staphylococcus aureus* (MRSA), extended-spectrum beta-lactamases (ESBL)-producing Enterobacteriaceae, *Pseudomonas aeruginosa*, and carbapenem-resistant *Acinetobacter baumannii*.^17,18^

An increase in antimicrobial resistance has already been reported in studies including adult and pediatric patients, mainly in underdeveloped or developing countries,^19^ and an increase in infections by MDR bacteria has also been observed in NICUs.^20,21,22,23^

Aiming to establish criteria for the different levels of resistance to antimicrobials and unify the identification and surveillance of the main bacteria associated with MDR (*K. pneumoniae*, *P. aeruginosa*, and *Acinetobacter* spp.), a consensus was created by the countries that comprise the Latin American Network for Antibiotic Resistance Surveillance, coordinated by the Pan-American Health Organization (PAHO). In this protocol, the bacteria, after being tested for predefined groups of antibiotics, can be classified according to the identified resistance as MDR, extended drug resistance (XDR), or pandrug resistance (PDR).^24^

The continuous surveillance of the most frequent infectious agents in NICUs and their resistance to antimicrobials is important to plan a more effective initial empirical therapy,^25^ and subsequently, bacterial identification by blood cultures allows drug de-escalation, reducing the risk of resistance and adverse events.^26^ Late-onset HAIs pose a risk to the neonate, intrahospital environment, and external intrapersonal and environmental contamination, with an increased risk if there are MDR bacteria.

The objective of this study was to describe the bacterial flora in a public NICU and their *in vitro* sensitivity to antimicrobials, aiming to identify the presence of MDR bacteria in neonatology.

## METHOD

This is a cross-sectional study on the identification and *in vitro* antimicrobial resistance of bacteria associated with late-onset HAIs with laboratory confirmation in the NICU of a public referral hospital in the state of Ceará, Brazil, from January 2013 to December 2017.

Hospital Geral Waldemar Alcântara is a secondary care hospital from the public health system, being the first public hospital in the North and Northeast regions of Brazil to receive the title of level 2 hospital facility awarded by the National Accreditation Organization. It supports the tertiary care network in the state of Ceará, attending exclusively the Brazilian Unified Health System users. It has 336 beds, distributed for internal medicine, surgical wards, pediatric wards, special care unit, adult intensive care unit, NICU, pediatric intensive care unit, and medium-risk nursery, in addition to outpatient and home care. The neonatology service has 8 NICU beds and 16 conventional neonatal intermediate care unit beds.

The study population consisted of NBs with late-onset bacterial HAI with laboratory confirmation by blood culture according to the standardized criteria and notified by the Hospital Infection Control Committee (CCIH, *Comissão de Controle de Infecção Hospitalar*) during the study period. Late-onset HAI of hospital origin was defined as the infection occurring after the first 48 h of life.^4^

**Figure 1.**
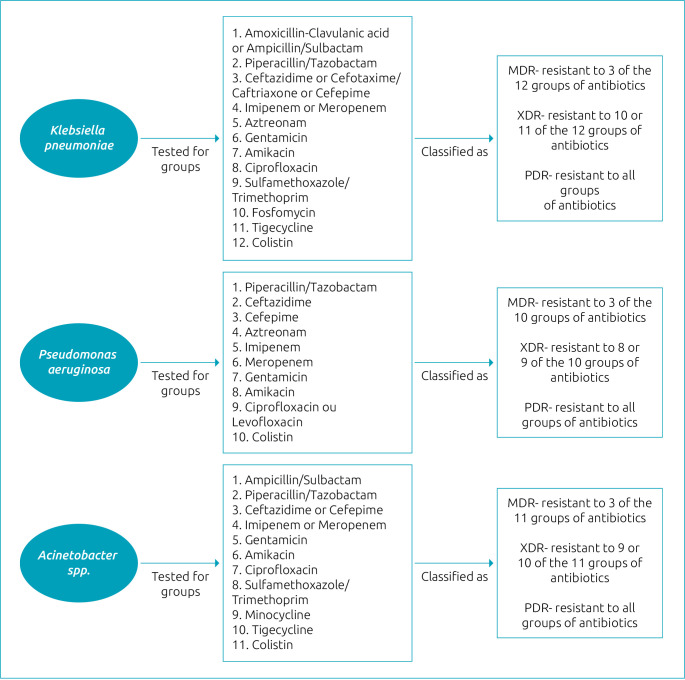
Evaluation of bacterial multidrug resistance according to the criteria of the Pan-American Health Organization:^24^ multidrug resistance (MDR), extended drug resistance (XDR), or pandrug resistance (PDR).

Data were collected from CCIH documents and medical records. The type of microorganism (Gram-negative or Gram-positive bacteria), the bacterial species, the sensitivity to the tested antimicrobials that can be used in the neonatal period, and the presence or absence of MDR bacteria were described. The isolation of the microorganisms was carried out by an automated method, and antimicrobial susceptibility testing (AST) was obtained from the microbiology laboratory linked to the hospital. The susceptibility to antimicrobials was defined according to the reference criteria: Clinical and Laboratory Standards Institute, with adaptations of Agência Nacional de Vigilância Sanitária (ANVISA) Technical Standard 01/2013.^27^ Both the intermediate and resistant antimicrobial criteria were considered non-susceptible. Gram-negative bacteria that were considered multiresistant by the hospital from January 2013 to December 2017 were evaluated in this study for resistance to all tested antimicrobials, regardless of whether or not they can be used in the neonatal period, and subsequently were reclassified according to the criteria adopted in 2019 by the PAHO^24^ as MDR, XDR, or PDR (Figure 1).

The categorical quantitative results were presented as percentages and counts in tables. The collected data were tabulated and analyzed using the IBM SPSS Statistics for Windows, version 23.0 software (IBM Corp., Armonk, NY, USA).

The study was approved by the Ethics Committee of the public hospital without the need of an informed consent form (protocol 61/2017; CAAE 0211.0.037.000-11).

## RESULTS

In the 5 years of study, the incidence rate of late-onset HAI was 10.7%, and the incidence density of late-onset HAI was 10.6 per 1,000 patients-day. Regarding the topography of the infections, 49.0% were cases of clinical sepsis with or without laboratory confirmation, 25.0% were cases of pneumonia, 12.0% were cases of necrotizing enterocolitis, and 3.0% were cases of central line-associated bloodstream infection, which are among the main ones.

Of the 427 patients reported as having late-onset HAIs, 54 (12.6%) had a positive blood culture record, and among the infections, 47 were caused by bacteria (11.0% positivity) and 7 by fungi (excluded from the analysis).

Among the types of isolated bacteria, 26 (55.3%) were Gram-negative and 21 (44.7%) were Gram-positive, of which 7 (14.9%) were classified as MDR: six Gram-negative bacteria and one Gram-positive bacteria (classified by the CCIH as MDR bacteria due to non-susceptibility to oxacillin and vancomycin).

Of the seven cases of infection caused by MDR bacteria, 4 (57.1%) were identified in female NBs and 3 (42.9%) in male NBs; the 4 NBs had birth weight <1,500 g (57.1%), and 3 NBs had birth weight between 1,500 g and 2,499 g (42.9%); 1 (14.3%) NB had <28 weeks of gestational age (GA) at birth, 5 (71.4%) NBs had ≥28 weeks of GA and <34 weeks of GA, and 1 (14.3%) NB had ≥34 weeks and <37 weeks of GA.

Of the 26 Gram-negative bacteria, 8 species were isolated: *K. pneumoniae* (23.4% [n=11]), *P. aeruginosa* (10.6% [n=5]), *E. coli* (8.5% [n=4]), and *Enterobacter cloacae* (4.3% [n=2]), followed by *Proteus mirabilis*, *Serratia marcescens*, *A. baumannii*, and *Enterobacter asburiae*, 2.1% each. Of the 21 Gram-positive species, 4 species were isolated: *Staphylococcus epidermidis* (21.3% [n=10]), *Staphylococcus haemolyticus* (10.6% [n=5]), *Enterococcus faecalis* (10.6% [n=5]), and *S. aureus* (2.1% [n=1]).

For Gram-negative bacteria, the susceptibility to amikacin, ampicillin, ampicillin/sulbactam, piperacillin/tazobactam, second- to fourth-generation cephalosporins (cefoxitin, ceftazidime, ceftriaxone, cefepime, cefuroxime), colistin, gentamicin, and carbapenem antimicrobials (ertapenem, imipenem, meropenem) was studied. Cephalosporins and carbapenems were pooled for the analysis. The drugs aztreonam, ciprofloxacin, sulfamethoxazole/trimethoprim, and tigecycline, which are not routinely used in neonates, were kept in the analysis for the classification of MDR.

Among the Gram-negative bacteria, 92.3% (n=24) showed resistance to more than one antimicrobial agent, the most common being ampicillin and cephalosporins. In relation to ampicillin, resistance was 81.2%, with an increase in response to the association with sulbactam (resistance of 58.0%). Regarding the other tested drugs, the greater resistance was to colistin, followed by gentamicin, piperacillin/tazobactam, and amikacin (Table 1).

**Table 1.** Microbial sensitivity of Gram-negative bacteria, identified from blood cultures in a public Neonatal Intensive Care Unit, to amikacin, ampicillin, ampicillin/sulbactam, colistin, gentamicin, and piperacillin/tazobactam, Ceará, Brazil, 2013–2017.

Bacterial species/number (n)	Sensitivity (%)					
	Amikacin	Ampicillin	Ampicillin/sulbactam	Colistin	Gentamicin	Piperacillin/tazobactam
*Klebsiella pneumoniae* (n=11)	90.9	0.0	36.4	100.0	54.5	72.7
Pseudomonas aeruginosa* (n=5)	40.0	0.0	0.0	100.0	40.0	40.0
*Escherichia coli* (n=4)	100.0	50.0	100.0	100.0	100.0	100.0
*Enterobacter cloacae* (n=2)	100.0	0.0	0.0	100.0	50.0	50.0
*Acinetobacter baumannii* (n=1)	100.0	0.0	100.0	100.0	100.0	100.0
*Serratia marcescens* (n=1)	100.0	0.0	0.0	0.0	100.0	100.0
*Proteus mirabilis* (n=1)	100.0	100.0	100.0	0.0	100.0	100.0
*Enterobacter asburiae* (n=1)	100.0	0.0	0.0	100.0	100.0	100.0
**Total (n=26)**	**91.4**	**18.8**	**42.0**	**75.0**	**80.6**	**82.8**

*One *P. aeruginosa* bacterium was not tested for ampicillin/sulbactam, and one was not tested for colistin.

Regarding cephalosporins, the greater resistance rates were to cefoxitin and cefuroxime, followed by ceftriaxone, ceftazidime, and cefepime (Table 2). Regarding ertapenem, resistance was only 3.0% among the tested bacteria. The *in vitro* response to meropenem and imipenem was similar, with resistance of 9.8% (Table 3).

**Table 2.** Microbial sensitivity of Gram-negative bacteria, identified from blood cultures in a public Neonatal Intensive Care Unit, to the second- to fourth-generation cephalosporins, Ceará, Brazil, 2013–2017.

Bacterial species/number (n)	Sensitivity (%)				
	Cefoxitin	Ceftazidime	Ceftriaxone	Cefepime	Cefuroxime
*Klebsiella pneumoniae* (n=11)	54.5	45.5	45.5	45.5	45.5
Pseudomonas aeruginosa* (n=5)	0.0	40.0	0.0	40.0	0.0
*Escherichia coli* (n=4)	100.0	100.0	100.0	100.0	100.0
*Enterobacter cloacae* (n=2)	0.0	50.0	50.0	50.0	50.0
*Acinetobacter baumannii* (n=1)	0.0	100.0	100.0	100.0	0.0
*Serratia marcescens* (n=1)	0.0	100.0	100.0	100.0	0.0
*Proteus mirabilis* (n=1)	100.0	100.0	100.0	100.0	100.0
*Enterobacter asburiae* (n=1)	0.0	0.0	0.0	0.0	0.0
**Total (n=26)**	**31.8**	**66.9**	**61.9**	**66.9**	**36.9**

*P. aeruginosa bacterium was not tested for cefoxitin, one was not tested for ceftriaxone, and one was not tested for cefuroxime.

**Table 3.** Microbial sensitivity of Gram-negative bacteria, identified from blood cultures in a public Neonatal Intensive Care Unit, to carbapenems, Ceará, Brazil, 2013–2017.

Bacterial species/ number (n)	Sensitivity (%)		
	Ertapenem	Imipenem	Meropenem
*Klebsiella pneumoniae* (n=11)	81.8	81.8	81.8
Pseudomonas aeruginosa* (n=5)	–	40.0	40.0
*Escherichia coli* (n=4)	100.0	100.0	100.0
*Enterobacter cloacae* (n=2)	100.0	100.0	100.0
Acinetobacter baumannii**(n=1)	–	100.0	100.0
*Serratia marcescens* (n=1)	100.0	100.0	100.0
*Proteus mirabilis* (n=1)	100.0	100.0	100.0
*Enterobacter asburiae* (n=1)	100.0	100.0	100.0
**Total (n=26)**	**97.0**	**90.2**	**90.2**

*Five *P. aeruginosa* bacteria were not tested for ertapenem. **One *A. baumannii* bacterium was not tested for ertapenem.

For Gram-positive bacteria, susceptibility to linezolid, oxacillin, penicillin, vancomycin, and teicoplanin was assessed. The following drugs were excluded from the analysis and discussion: clindamycin, erythromycin, ciprofloxacin, moxifloxacin, norfloxacin, rifampicin, sulfamethoxazole/trimethoprim, and tigecycline, which are not routinely used in neonates, in addition to gentamicin, which is not the first choice for Gram-positive microorganisms.

Among the Gram-positive bacteria, similar non-susceptibility was found between linezolid and teicoplanin (25.0%), with approximately half of the strains being resistant to oxacillin (47.5%), with high resistance to penicillin (80.0%). Vancomycin resistance was found in 2.5% (Table 4).

**Table 4.** Microbial sensitivity of Gram-positive bacteria, identified from blood cultures in a public Neonatal Intensive Care Unit, to routinely used antimicrobials, Ceará, Brazil, 2013–2017.

Bacterial species/ number (n)	Sensitivity (%)				
	Linezolid	Oxacillin	Penicillin	Vancomycin	Teicoplanin
*Staphylococcus epidermidis* (n=10)	100.0	50.0	0.0	90.0	100.0
*Staphylococcus haemolyticus* (n=5)	100.0	60.0	0.0	100.0	100.0
*Enterococcus faecalis* (n=5)	100.0	100.0	80.0	100.0	100.0
*Staphylococcus aureus* (n=1)	0.0	0.0	0.0	100.0	0.0
**Total (n=21)**	**75.0**	**52.5**	**20.0**	**97.5**	**75.0**

In relation to MDR in Gram-negative bacteria, all tested antimicrobials, listed in the study published by PAHO,^24^ were evaluated, and among the six Gram-negative MDR bacteria, three samples were of *K. pneumoniae* and three of *P. aeruginosa*, reclassified as two MDR and one XDR, and three XDR, respectively. Among the *K. pneumoniae* bacteria, the resistance rates found to the antimicrobials used in the NICU were in decreasing order: 100.0% to ampicillin, 63.6% to ampicillin/sulbactam, 52.7% to cephalosporins, 45.5% to gentamicin, 27.3% to piperacillin/tazobactam, 18.2% to imipenem and meropenem, and 9.1% to amikacin. For *P. aeruginosa*, resistance rates were 60.0% to all tested antimicrobials from the protocol list, except to colistin, to which the microorganism did not show resistance among those tested (Tables 1 to 3).

Regarding the outcome, of the seven cases of infection caused by MDR bacteria, one NB, with 31 weeks of GA and birth weight of 1,985 g, died (14.3% of the MDR ones). Treatment was started on the 23rd day of life with meropenem and vancomycin, after previous treatment with piperacillin/tazobactam. When *P. aeruginosa* was isolated, the microorganism was sensitive only to Polymyxin-B. Death occurred at 34 days of life, and the reported cause of death was neonatal sepsis.

## DISCUSSION

This study showed that the occurrence of late-onset bacterial HAIs with laboratory confirmation was 11.0%; therefore, most infections were diagnosed using clinical and laboratory criteria without confirmation by blood culture. Among the positive blood cultures, the predominant agents in this study were Gram-negative bacteria.

Among the Gram-negative bacteria, the resistance rates among the most frequently used antimicrobials in the NICU were ceftriaxone (38.1%), ceftazidime and cefepime (33.1% both), gentamicin (19.4%), and piperacillin/tazobactam (17.2%). These findings highlight the need for the rational use of cephalosporins in general, which are widely used in NICUs for late-onset infections. Overall, resistance to amikacin was 8.6%, making this drug a better therapeutic option than gentamicin. Ampicillin and ampicillin/sulbactam are not good empirical therapeutic options. In this hospital, the first choice of empirical treatment for late-onset HAI is piperacillin/tazobactam, while awaiting the culture results. In this study, the lower resistance found to piperacillin/tazobactam supports its empirical use *versus* cephalosporins. In case of therapeutic failure, the antimicrobial used in the service is meropenem, either alone or in combination with vancomycin. Vancomycin is initiated depending on the culture results or if an infectious focus is suspected on the skin or associated with invasive devices.

Resistance to carbapenems ranged from 3.0 to 9.8%, which makes these drugs good options in cases of therapeutic failure. Although resistance to colistin was 25.0%, this fact was due to resistant *S. marcescens* and *P. mirabilis* strains, with no resistance being found to the other evaluated species, which makes it still an option for more complex cases.

Among *K. pneumoniae* strains, resistance to piperacillin/tazobactam was 27.3%, whereas it was 18.2% to imipenem and meropenem, indicating a more resistant profile in relation to other Gram-negative bacteria (Tables 1 and 3), except for *P. aeruginosa*, which is resistant to 60.0% of the antimicrobials. In the presence of these infectious agents, it is necessary to observe the results of cultures and clinical evolution of the NB more stringently, due to the greater antimicrobial resistance.

Late-onset HAI was addressed in two systematic reviews in NICUs,^23^ where *Klebsiella*, *Acinetobacter,* and *Pseudomonas* were the main pathogens, with 61.1–85.6% resistance to ceftriaxone and cefotaxime, much higher than the results of this study. The results were approximate in this study in relation to resistance to other antimicrobials, except for *Pseudomonas*: moderate resistance to piperacillin/tazobactam and low resistance to imipenem were observed.

The comparison of our results with the study of 230 blood cultures positive for Gram-negative bacteria by Patel et al.^25^ showed that the most common isolated species were *K. pneumoniae* (23% in both studies), *P. aeruginosa* (10.6% [n=5] in our study *versus* 6.5% [n=15] in the other study), and *E. coli* (6.4% [n=3] in our study and 30.4% [n=70] in the other study). *Escherichia coli* was the main Gram-negative agent reported by Patel et al.^25^

In the study by Patel et al.,^25^ 23.0% of bacteria were generally resistant to at least one of the tested antimicrobials, a much lower rate than the one found in this study, in which when combining overall resistance of all Gram-negative bacteria, 92.3% were resistant to more than one antimicrobial. When comparing the results, resistance to gentamicin was 19.4% *versus* 14.8% in the aforementioned study, whereas the resistance rates of piperacillin/tazobactam were much higher, 17.2% *versus* 9.9%; among cephalosporins, resistance rates ranged from 33.1 to 68.2% *versus* the 7.0% found for the third-generation cephalosporins; and of the three carbapenem agents, a resistance of 7.5% *versus* 4.5% was observed.^25^

Other studies also observed a predominance of infections by Gram-negative bacteria in an Indian study (61.0% of cases)^28^ and in a study carried out in Jordan (62.0%).^29^ Regarding drug resistance, in a study carried out by Yusef et al.,^29^ a high rate of bacterial multiresistance, of about 39.0%, was observed, which was much higher than that observed in this study, which was 14.9%. In a multicenter study carried out in Brazil and Italy,^30^ a 45.0% rate of MDR bacteria was found, but the study included pediatric patients, highlighting the need for more specific studies for the neonatal period such as this one.

The resistance of Gram-positive bacteria to penicillin was the most common (80.0%), followed by resistance to oxacillin (47.5%). Resistance to vancomycin is low (2.5%), although the finding of a Gram-positive bacteria resistant to oxacillin and vancomycin warns for the risk of bacterial resistance to the latter drug.

For the empirical treatment of Gram-positive bacteria, with approximately 50.0% of the bacteria being resistant to oxacillin, the therapeutic option of linezolid and teicoplanin remains, with 25.0% of resistance each. In case of therapeutic failure or in more complex cases, vancomycin is another option since resistance to it is low (2.5%). Resistance to vancomycin is concerning because there are a few therapeutic options approved for neonates. In this hospital, vancomycin is used upon suspected infection by Gram-positive bacteria, while awaiting the culture results.

Although the results found in this study are consistent with the national and international literature, there are noteworthy limitations. The use of secondary data could retrospectively lead to incomplete records, which were bypassed by requesting blood culture results from the laboratory, which were not recorded in the hospital medical records service. Moreover, we have a low number of bacterial isolation cultures, possibly caused by the practice of collecting only one blood culture sample in neonates.

Despite the limited number of positive blood cultures in this study, knowledge of the bacterial flora and its pattern of antimicrobial resistance can guide rational antibiotic use, preventing unnecessary or excessive use. This study can and should be reproduced in other services, with the purpose of planning institutional protocols and individualized treatments, when needed.

A low number of positive blood cultures was observed and that could be improved by collecting two samples for blood cultures in neonates, which is not a routine practice in some services, as verified in this study. We suggest that the NICU services consider the possibility of collecting two samples for blood cultures whenever the patient’s clinical conditions allow it.

We also suggest the development of a specific MDR classification for the neonatal period by the competent authorities, since some antimicrobials listed in the consensus published by PAHO, such as aztreonam, ciprofloxacin, fosfomycin, levofloxacin, sulfamethoxazole/trimethoprim, and tigecycline, are not routinely used in NBs.

In this study, infections were more frequent in NBs in the low birth weight and preterm groups (85.7%, with <34 weeks of GA), which demonstrates the importance of continuous surveillance of those who are more frequently submitted to invasive procedures and/or prolonged stay in the NICU. The death of one NB caused by a late HAI by *Pseudomonas*, susceptible only to Polymyxin-B, endorses the implementation of new strategies.

This study emphasized the importance of continuous monitoring of the local bacterial flora and its response to commonly used antimicrobials, aiming to avoid therapeutic failure, stressing that rational antimicrobial use can prevent the emergence of MDR bacteria. The finding of MDR bacteria in this study, including bacteria classified as extended resistance, reinforces the importance of strategies aimed to prevent late-onset HAIs and the rational use of antimicrobials.

## Data Availability

The database that originated the article is available with the corresponding author.
